# Macroinvertebrate identity mediates the effects of litter quality and microbial conditioning on leaf litter recycling in temperate streams

**DOI:** 10.1002/ece3.3790

**Published:** 2018-02-04

**Authors:** Mathieu Santonja, Laura Pellan, Christophe Piscart

**Affiliations:** ^1^ Université Rennes 1 – UMR CNRS 6553 ECOBIO Rennes France

**Keywords:** feces production, freshwater macroinvertebrates, functional trait, litter consumption, temperate stream

## Abstract

Plant litter decomposition is an essential ecosystem function that contributes to carbon and nutrient cycling in streams. Aquatic shredders, mainly macroinvertebrates, can affect this process in various ways; they consume leaf litter, breaking it down into fragments and creating suitable habitats or resources for other organisms through the production of fine particulate organic matter (FPOM). However, measures of litter‐feeding traits across a wide range of aquatic macroinvertebrates are still rare. Here, we assessed the contributions of 11 species of freshwater macroinvertebrates to litter decomposition, by measuring consumption rate, FPOM production, and assimilation rate of highly decomposable (*Alnus glutinosa*) or poorly decomposable (*Quercus robur*) leaf litter types. In general, an increase in the quality of litter improved the litter consumption rate, and fungal conditioning of the leaf litter increased both the litter consumption rate and FPOM production. Macroinvertebrates specializing in leaf litter consumption also appeared to be the most sensitive to shifts in litter quality and the conditioning process. Contrary to expectations, the conditioning process did not increase the assimilation of low‐quality litter. There was a strong correlation between the relative consumption rate (RCR) of the two litter types, and the relative FPOM production (RFP) was strongly correlated to the RCR. These findings suggest a consistent relationship between RCR and macroinvertebrate identity that is not affected by litter quality, and that the RFP could be inferred from the RCR. The varying responses of the macroinvertebrate feeding traits to litter quality and the conditioning process suggest that the replacement of a shredder invertebrate species by another species could have major consequences for the decomposition process and the detritus‐based food web in streams. Further studies onto the importance of invertebrate identity and the effects of litter quality in a variety of freshwater ecosystems are needed to understand the whole ecosystem functioning and to predict its response to environmental changes.

## 1 INTRODUCTION

Leaf litter decomposition is an essential ecosystem function controlling the carbon and nutrient cycles in both terrestrial and aquatic ecosystems (Cadish & Giller, 1997; Gessner et al., 2010; Handa et al., 2014). Litter decomposition is affected by the litter quality, namely the physical and chemical properties of plant litter (Cornelissen, 1996; Cornwell et al., 2008; Coûteaux, Bottner, & Berg, 1995), environmental conditions such as temperature (Fierer, Craine, McLauchlan, & Schimel, 2005; Hobbie, 1996), and the detrital food web structure (Garcia‐Palacios, McKie, Handa, Frainer, & Hättenschwiler, 2016; Hättenschwiler, Tiunov, & Scheu, 2005).

Even if the decomposition process involves both physical (hydrologic fractioning) and biological factors (namely microbial decomposition and invertebrate shredding activities), the role played by shredder macroinvertebrates remains essential in quantitative terms (Anderson & Sedell, 1979; Cummins & Klug, 1979; Garcia‐Palacios et al., 2016; Handa et al., 2014). These macroinvertebrates contribute directly or indirectly to litter decomposition by consuming and fragmenting litter material (Allan, 1996; Graça, 2001), providing additional nutrients and habitats for microbes, and creating new resources for other organisms (collectors and filter‐feeding invertebrates) in the aquatic food web through the production of fine particulate organic matter (FPOM) (Hall, Wallace, & Eggert, 2000; Joyce & Wotton, 2008; Wallace, Eggert, Meyer, & Webster, 1997; Wetzel, 1995). However, there is considerable variation in litter consumption and FPOM production among macroinvertebrates (Dangles & Malmqvist, 2004; Friberg & Jacobsen, 1994; Piscart, Mermillod‐Blondin, Maazouzi, Mérigoux, & Marmonier, 2011) and according to decomposition stage of the litter (Foucreau, Puijalon, Hervant, & Piscart, 2013).

As the composition of the macroinvertebrate community strongly affects litter decomposition rates, it is important to identify and measure the key feeding traits of dominant macroinvertebrates. Measuring the effect of such traits in a range of macroinvertebrate species of different sizes and specialization levels on litter consumption may improve the understanding of the extent to which macroinvertebrates actually contribute to litter decomposition. Furthermore, if a change in environmental conditions leads to a change in the macroinvertebrate community structure, it might be possible to predict how these changes could affect litter decomposition. Surprisingly, while leaf plant and terrestrial macroinvertebrate traits are rather well known for many species (Cornwell et al., 2008; Kattge et al., 2011; Moretti et al., 2017) and are available in several trait databases, very little information on aquatic macrodetritivore traits are available in literature. More precisely, there is a lack of information about the factors influencing the consumption of leaf litter for most aquatic macroinvertebrates. Published studies only focus on few species, and when a higher number of species is used, they are generally closely related species (Bjelke & Herrmann, 2005; Piscart et al., 2011). As the link between leaf litter and macroinvertebrate is species‐specific, reliable measures of their feeding traits could help (1) to better understand litter decomposition process and (2) to predict in which extent shifts in plant and/or macroinvertebrate communities could alter the decomposition process.

To address this gap, this study assessed the contributions of 11 species of aquatic macroinvertebrates to litter decomposition by measuring their daily leaf consumption and FPOM production rates, two feeding traits that could significantly affect litter decomposition. More specifically, this study tested (1) whether macroinvertebrate species differ in their litter‐feeding traits; and how their feeding traits changes according (2) to the litter quality and (3) to the leaf fungal conditioning. To answer these questions, a highly decomposable alder leaf litter (*Alnus glutinosa*, hereafter referred to as *Alnus*) and a poorly decomposable oak leaf litter (*Quercus robur*, hereafter referred to as *Quercus*) (Cornelissen, 1996; Foucreau, Puijalon, et al., 2013) were selected. These were tested under laboratory conditions to examine the effect of both the type of leaf and the effect of the conditioning process on leaf consumption, FPOM production, and the assimilation rate of the 11 macroinvertebrate species.

Firstly, we hypothesized that, among the feeding traits, the litter consumption rate is mainly driven by the shredder identity, whereas the assimilation rate is mainly controlled by the litter quality. Secondly, we hypothesized that the microbial conditioning would increase both the consumption and assimilation rates by increasing the litter quality. Thirdly, it was predicted that the effects of the conditioning process would be higher in *Quercus* litter compared to *Alnus* litter, as *Alnus* litter is already a highly decomposable leaf litter.

## 2 MATERIALS AND METHODS

### 2.1 Collection of aquatic macroinvertebrates

A total of 11 aquatic macroinvertebrate species from different taxonomic groups (Crustacea, Insecta, Gastropoda) were chosen in order to ensure a good representation of the taxonomic, functional, and size diversities of European freshwater shredder macroinvertebrates (Tachet, Richoux, Bournard, & Usseglio‐Polatera, 2010; Table 1). Among the taxonomic groups, only the species with the highest affinity for the modalities “Shredder” for the feeding habits and “Plant detritus ≥ 1 mm” for the food were selected according to Tachet et al. (2010).

**Table 1:** Macroinvertebrate species characteristics. Scientific name and associated Order, code used in the article, location where the species were collected, mean dry weight (±*SE*), and specialization index for leaf litter consumption (calculated following the Grinnellian specialization index; Devictor et al., 2010) are indicated

Species	Order	Code	Location	Dry weight (mg)	Specialization
*Planorbarius corneus* (Linnaeus, 1758)	Gastropoda	PLCO	Couesnon	13.12 ± 0.73	0.65
*Crangonyx pseudogracilis* Bouesfiled, 1958	Amphipoda	CRPS	Vilaine	1.54 ± 0.08	1.73
*Echinogammarus berilloni* (Catta, 1878)	Amphipoda	ECBE	Hermitage	4.26 ± 0.25	0.71
*Gammarus pulex* (Linnaeus, 1758)	Amphipoda	GAPU	Geuche	5.79 ± 0.48	0.71
*Gammarus tigrinus* Sexton, 1939	Amphipoda	GATI	Vilaine	2.60 ± 0.19	0.71
*Asellus aquaticus* (Linnaeus, 1758)	Isopoda	ASAQ	Apigné	4.02 ± 0.24	1.25
*Chaetopteryx villosa* (Fabricius, 1798)	Trichoptera	CHVI	Hermitage	3.77 ± 0.38	1.40
*Halesus radiatus* (Curtis, 1834)	Trichoptera	HARA	Hermitage	25.94 ± 1.93	1.07
*Lepidostoma hirtum* (Fabricius, 1775)	Trichoptera	LEHI	Selune	1.64 ± 0.16	2.09
*Limnephilus flavicornis* (Fabricius, 1787)	Trichoptera	LIFL	Hermitage	10.83 ± 0.61	1.40
*Sericostoma personatum* (Kirby and Spence, 1826)	Trichoptera	SEPE	Everre	10.45 ± 0.85	0.94

The individuals were collected from four streams, and three rivers located around the city of Rennes in April 2016 (Table 2). The temperature, electrical conductivity at 25°C, and dissolved oxygen concentration were recorded at each site (Table 2) during macroinvertebrate samplings using a portable apparatus (Odeon, Ponsel Mesure, France). Following the samplings, each species was maintained separately at 12°C (a temperature close to that of the stream water) in 5‐L tanks filled with filtered (GF/C, 1.2 mm pore size; Whatman, UK) and aerated water from their own site under a 12:12‐hr light:dark regime for 48 hr. During this period, the animals were kept without food to empty their gut.

**Table 2:** Location and main physicochemical characteristics of the sites where the eleven macroinvertebrate species were collected

	Vilaine river	Selune river	Apigné stream	Everre stream	Hermitage stream	Couesnon river	Geuche stream
Location	47°34′N	48°08′N	48°09′N	48°18′N	48°28′N	48°30′N	48°38′N
	2°02′W	1°17′W	−1°74′W	1°24′W	1°33′W	−1°30′W	1°00′W
Temperature (°C)	11.6–12.8	10.0–10.3	12.9–13.1	10.0–10.5	9.9–10.6	9.6–9.8	7.9–8.8
Conductivity (S/m)	250–257	198–209	108–476	221–229	132–139	230–277	199–200
Dissolved oxygen (mg/L)	10.6–11.0	10.5–10.6	5.0–8.4	11.1–12.2	10.0–11.3	6.4–7.1	10.6–10.8

### 2.2 Experimental setup

#### 2.2.1 Pretreatment for fungal conditioning of leaf litter

Freshly abscised leaves of *Alnus* and *Quercus* were collected during the period of maximal litter fall from October to November 2015. Immediately after collection, the leaves were air dried and stored at room temperature. “Unconditioned leaves” were stored at room temperature until the beginning of the experiment, while “conditioned leaves” were obtained by immersing leaves into a stream to allow microbial conditioning. Similarly sized leaves of the same species were enclosed in fine mesh litterbags (0.5‐mm mesh), which excluded most macroinvertebrates and allowing microbial colonization (Boulton & Boon, 1991). The litterbags were immersed in the Hermitage stream, which is surrounded by deciduous woodland (48°28′ N, 1°33′W; Piscart, Genoel, Dolédec, Chauvet, & Marmonier, 2009). The litterbags containing *Alnus* leaves were collected after 3 weeks, and those containing *Quercus* leaves were collected after 9 weeks of field exposure in order to allow fungal conditioning of leaf litter. For the two tree species, the conditioning time (3 or 9 weeks) corresponded to the estimated number of days needed for a peak of fungal colonization (see Foucreau, Puijalon, et al. (2013) for more details).

In the laboratory, unconditioned and conditioned leaves were cut into 10‐mm diameter disks, avoiding the central veins, air dried for four hours to preserve the effect of microbial conditioning, and weighed by three‐disk pack. For each type of leaf (i.e., both unconditioned and conditioned leaves of *Alnus* and *Quercus*), 30 randomly selected disks were then dried at 65°C for 72 hr and weighed to determine the water content remaining after air drying. The water content (comprising between 0.12% and 0.33% of the disk weight) was used to correct the initial weight of the air‐dried leaf disks used in the experiment.

#### 2.2.2 Leaf consumption by macroinvertebrates

A total of 10 individuals of each macroinvertebrate species for each litter × conditioning type combination were placed in individual microcosms (7 cm diameter) filled with 30 ml of filtered water from their own site for a total of 440 microcosms (11 macroinvertebrate species × 2 leaf litter species × 2 conditioning types × 10 replicates). Three weighed disks (±0.1 mg) were added to each microcosm to reduce any variability caused by between‐leaf differences in thickness, hardness, or colonization. The remaining leaf materials were checked every day, and a new weighted disk was added when a disk was completely consumed in order to maintain the same quantity of food in the microcosm. To account for the litter mass loss due to microbial decomposition or/and leaching, a supplementary treatment without macroinvertebrates was added for each litter species × conditioning type combination. This was treated in the same way as the other treatments for a total of 84 control microcosms (7 water sites × 2 litter species × 2 conditioning types × 3 replicates). The 524 microcosms (440 with macroinvertebrates + 84 without macroinvertebrates) were placed in a climate‐controlled room at 12°C, with 80% humidity and a 12:12‐hr light:dark regime. The microcosms were not agitated or aerated during the experiment. Dissolved oxygen concentrations were randomly measured every day, and no strong depletion in the oxygen content of the water was recorded.

After 3 days, the remaining leaf material and the particles up to 1 mm were hand collected, dried at 65°C for 72 hr, and weighed to the nearest 0.1 mg. The leaf consumption rate by macroinvertebrate was calculated as the difference between the initial and final dry leaf mass minus the leaf mass loss due to microbial decomposition or/and leaching (i.e., corrected with the treatment without macroinvertebrate).

#### 2.2.3 Fine particulate organic matter production by macroinvertebrates

All individuals were kept in the microcosm and starved for 48 hr to empty their gut contents after the feeding experiment. Thereafter, macroinvertebrates were dried at 65°C for 72 hr and weighed in order to obtain the dry weight. The remaining water in each microcosm was then filtered through a previously weighed (AFDW) Whatman GF/C 1.2‐μm filter in order to measure any particles less than 1 mm in diameter. To perform this step, the filters were dried at 65°C for 72 hr and then weighed. The difference between the weight of the filter before and after filtration was calculated in order to obtain the dry weight of feces of each macroinvertebrate, corresponding to the fine particulate organic matter (FPOM) produced by macroinvertebrates during the 3‐day experiment and during the subsequent 2 days of starvation.

### 2.3 Leaf litter quality measurements

Leaf litter quality was determined from four samples of each of the four litter types (i.e., both conditioned and unconditioned leaves of *Alnus* and *Quercus*). The organic carbon (C) and total nitrogen (N) contents of leaves were determined by thermal combustion using a Flash EA 1112 series C/N elemental analyzer (Agilent, Santa Clara, CA, USA). The concentrations of lignin, cellulose, hemicellulose, and water‐soluble compounds (WSC) were determined according to the van Soest extraction protocol (Van Soest & Wine, 1967) using a fiber analyzer (Fibersac 24; Ankom, Macedon, NJ, USA). Phenolic concentrations were measured colorimetrically using the method of Santonja, Fernandez, Gauquelin, and Baldy (2015) with gallic acid as a standard.

### 2.4 Statistical analyses

All statistical analyses were carried out using the R software (R Core Team, 2013).

The differences in leaf litter chemical characteristics were assessed using one‐way ANOVAs, followed by Tukey tests to carry out posthoc pairwise comparisons.

The leaf consumption and FPOM production were calculated per mg of individual dry weight (i.e., mg leaf (or mg FPOM) mg macroinvertebrate^−1^ day^−1^) and termed the “relative consumption rate” (hereafter RCR) and the “relative FPOM production” (hereafter RFP). The assimilation rate (hereafter AR) was also calculated based on the percentage of consumed leaves that was not transformed as FPOM for other trophic levels. A general linear model approach was used to test for the effects of macrodetritivore species (separated in species identity (11 species) and body mass (continuous variable)), litter type (*Alnus* and *Quercus*) and litter conditioning level (unconditioned and conditioned leaves) on RCR, RFP, and AR.

Finally, simple linear regressions on log‐transformed data (Log *X + 1*) were performed to explore the relationships between RCR, RFP, AR, the specialization index in leaf litter consumption, and the improvement of RCR (or RFP) due to fungal conditioning. Using the diet data for the 11 macroinvertebrates from Tachet et al. (2010), a specialization index for leaf litter consumption was calculated following the Grinnellian specialization index (Devictor et al., 2010). The Grinnellian specialization index of a given species is described by its variance in performance across a given range of resources. The improvement of RCR (or RFP) was calculated as the difference between RCR (or RFP) on conditioned and unconditioned leaves.

## 3 RESULTS

### 3.1 Leaf litter quality

The chemical characteristics of leaves varied significantly according to the type of leaf (Table 3). *Alnus* litter exhibited a higher N concentration, lower C:N ratio, lower phenolic concentration, and lower lignin concentration than *Quercus* litter (Table 3). For both litter types, the chemical characteristics also changed according to the conditioning process (Table 3). Microbial conditioning led to a decrease in the C concentration in *Alnus* leaf litter, an increased N concentration in *Quercus* litter, and a decreased in the C:N ratio for both litter types (Table 3). Microbial conditioning (and/or leaching) decreased the concentrations of water‐soluble compounds and phenolics and, in contrast, increased the concentrations of lignin and cellulose (only for *Alnus*) (Table 3).

**Table 3:** Main initial leaf litter characteristics of the two species. Values are mean ± standard error (*SE*). WSC = water‐soluble compound. One‐way ANOVAs were performed for differences among species. *F*‐values and associated *p*‐values (with the respective symbols *p <* *.05, **p <* *.01, and ***p <* *.001) are indicated. Different letters denote significant differences among species, a < b < c < d (posthoc Tukey tests results)

	*Alnus glutinosa*		*Quercus robur*		One‐way ANOVA
	Unconditioned	Conditioned	Unconditioned	Conditioned	
Carbon (%)	46.41 ± 0.06b	33.79 ± 0.58a	47.49 ± 0.07b	43.53 ± 0.42b	301.40***
Nitrogen (%)	2.79 ± 0.02c	2.76 ± 0.05c	1.00 ± 0.02a	1.36 ± 0.04b	701.28***
Lignin (%)	11.37 ± 0.37a	25.46 ± 1.56c	17.27 ± 0.89b	27.46 ± 2.07c	29.07***
Cellulose (%)	14.81 ± 0.53a	21.14 ± 1.78b	23.25 ± 0.73b	24.67 ± 1.74b	10.78**
Hemicellulose (%)	26.04 ± 1.00b	25.94 ± 2.63b	22.38 ± 1.72ab	17.42 ± 2.20a	4.19*
WSC (%)	47.78 ± 0.91c	27.47 ± 1.51a	37.11 ± 1.00b	30.44 ± 1.78a	44.53***
Phenolics (%)	4.91 ± 0.36c	1.20 ± 0.11a	7.96 ± 0.04d	2.90 ± 0.11b	217.66***
C:N ratio	16.66 ± 0.08b	12.24 ± 0.02a	47.48 ± 0.82d	32.07 ± 0.69c	886.99***

### 3.2 Relative consumption rate

**Figure 1:**
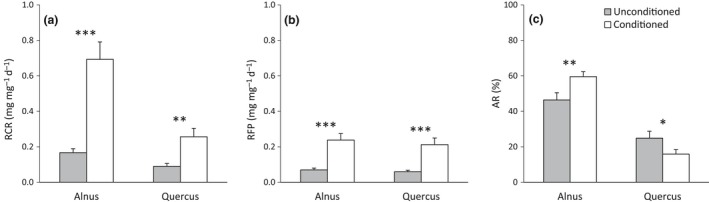
Mean values (±*SE*) of the (a) relative consumption rate, (b) relative FPOM production, and (c) assimilation rate according to the two litter types and the two conditioning levels. Significant differences according to the conditioning level are indicated with the respective symbols *p <* *.05, **p <* *.01, and ***p <* *.001

The relative consumption rate (RCR) significantly differed according to macroinvertebrate species (Table 4), from 0.11 to 1.03 mg leaf mg macroinvertebrate^−1^ day^−1^. RCR also decreased as macroinvertebrate mass increased (Table 4), although macroinvertebrate mass accounted for 15 times less of the overall variance in RCR than the “species effect” (Table 4). The RCR was three times higher for *Alnus* litter than *Quercus* litter (Table 4, Figure 1a) and was higher on conditioned leaves compared to unconditioned ones (Table 4, Figure 1a). However, the conditioning effect differed between the two litter types (significant litter type × litter conditioning interaction, Table 4), as the RCR was four times higher on conditioned *Alnus* leaves and only three times higher on conditioned *Quercus* leaves compared to unconditioned ones (Figure 1a).

**Table 4:** Output of general linear models testing for the effects of macrodetritivore species (separated in species identity and body mass), litter type, and litter conditioning level on relative consumption rate, relative FPOM production, and assimilation rate. *df* = degrees of freedom, %SS =  percentage of sums of squares. *F*‐values and associated *p*‐values (with the respective symbols *p <* *.05, **p <* *.01, and ***p <* *.001) are indicated

	*df*	Relative consumption rate		Relative FPOM production		Assimilation rate	
		%SS	*F*‐value	%SS	*F*‐value	%SS	*F*‐value
Detritivore species (DS)	10	24.2	18.4***	35.3	30.9***	6.6	3.8***
Detritivore mass (DM)	1	1.7	12.8***	1.4	12.5***	0.0	0.2
Litter type (LT)	1	5.8	44.4***	0.3	3.0	23.8	136.2***
Litter conditioning (LC)	1	9.5	72.5***	11.0	96.2***	0.1	0.6
DS × LT	10	4.2	3.2***	1.8	1.6	9.0	5.1***
DM × LT	1	0.0	0.2	0.2	1.9	0.1	0.4
DS × LC	10	12.2	9.3***	16.4	14.4***	3.0	1.7
DM × LC	1	1.1	8.7**	1.0	9.0**	0.1	0.3
LT × LC	1	2.5	19.4***	0.1	0.6	2.5	14.5***
DS × LT × LC	10	2.8	2.2*	1.1	0.9	7.2	4.1***
DM × LT × LC	1	0.2	1.2	0.0	0.0	0.0	0.0
Residuals	273	35.8		31.3		47.6	

**Figure 2:**
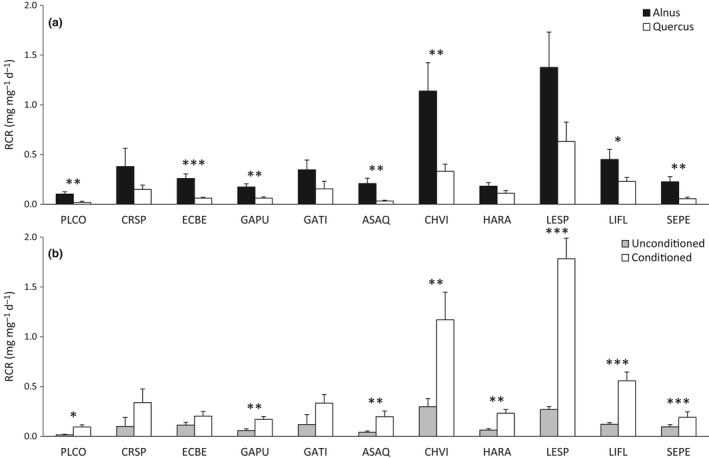
Mean values (±*SE*) of relative consumption rate of the 11 macroinvertebrate species according to (a) the two litter types and (b) the two conditioning levels. Significant differences for each macroinvertebrate species according to the litter type or the conditioning level are indicated with the respective symbols *p <* *.05, **p <* *.01, and ***p <* *.001

CHVI and LESP, belonging to the Trichoptera order, exhibited the highest RCR for each litter type and conditioning level (Figure 2a,b). As indicated by the significant macroinvertebrate species × litter type interaction, species‐specific RCR varied strongly depending on the litter type (Table 4, Figure 2a). Indeed, some macroinvertebrate species (CRSP, GATI, HARA) consumed *Alnus* and *Quercus* leaves at similar rates, whereas some other species (ASAQ, PLCO) consumed up to six times more *Alnus* than *Quercus* leaves (Figure 2a). In addition, some macroinvertebrate species (CRSP, ECBE, SEPE) consumed similar quantities of unconditioned and conditioned leaves, while some others (LESP, LIFL, PLCO) consumed up to seven times more conditioned than unconditioned leaves (significant detritivore species × litter conditioning interaction, Table 4, Figure 2b).

### 3.3 Relative FPOM production

**Figure 3:**
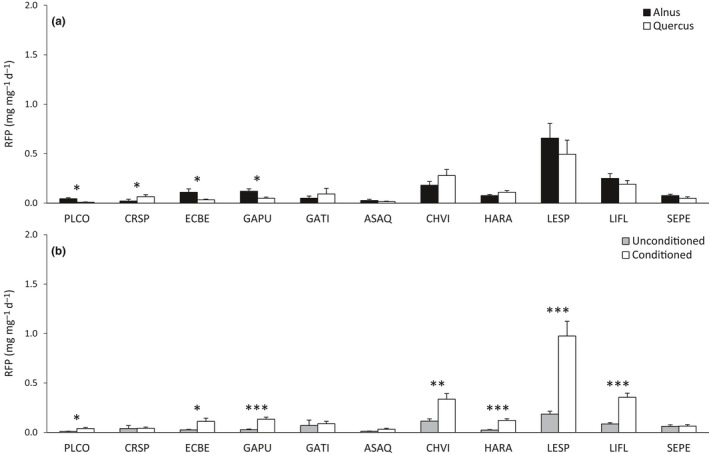
Mean values (±*SE*) of the FPOM production rate of the 11 macroinvertebrate species according to (a) the two litter types and (b) the two conditioning levels. Significant differences for each macroinvertebrate species according to the litter type or the conditioning level are indicated with the respective symbols *p <* *.05, **p <* *.01, and ***p <* *.001

The relative FPOM production (RFP) significantly varied among macroinvertebrate species (Table 4) and decreased as macroinvertebrate mass increased (Table 4). However, similar to RCR, macroinvertebrate mass explained 25 times less of the overall variance in RFP than did macroinvertebrate species identity (Table 4). RFP was marginally affected by the litter type (Table 4), because only three species (ECBE, GAPU, PLCO) exhibited a higher RFP with *Alnus* than with *Quercus* and, in opposite, one species (CRSP) exhibited a higher RFP with *Quercus* than with *Alnus* (Figure 3a). The RFP was three times higher for conditioned leaves compared to unconditioned ones (Table 4, Figure 1b). However, the extent to which litter conditioning affected species‐specific RFP varied (significant detritivore species × litter conditioning interaction, Table 4, Figure 3b). As for the RCR, the RFP of several species (ASAQ, CRSP, GATI, SEPE) was not increased by fungal conditioning, while the RFP of other species (GAPU, HARA, LESP) was up to five times higher on conditioned leaves compared to unconditioned ones (Figure 3b).

### 3.4 Assimilation rate

The assimilation rate (AR) varied from 25.5% to 56.6% depending on the macroinvertebrate species (Table 4). In contrast to RCR and RFR, AR was affected to a much greater extent by the litter type than by the macroinvertebrate species identity (Table 4), as the type of litter explained four times more of the overall variance in AR than did the macroinvertebrate species identity (Table 4). AR was two times higher on *Alnus* litter compared to *Quercus* litter (Table 4) and was not directly affected by litter conditioning (Table 4). The conditioning effect varied between the two litter types (significant litter type × litter conditioning interaction, Table 4), as AR increased on conditioned *Alnus* leaves and, in contrast, AR decreased in conditioned *Quercus* leaves compared to unconditioned ones (Figure 1c).

### 3.5 Relationships between RCR, RFP, and AR

**Figure 4:**
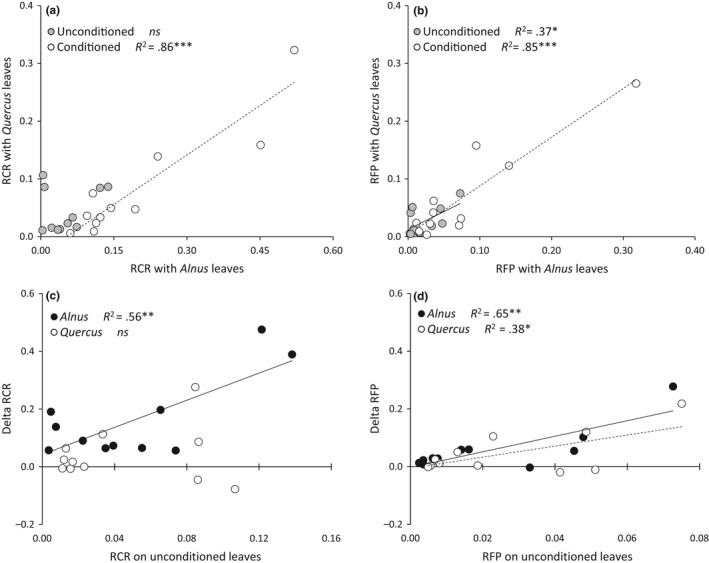
Relationships between (a) RCR of two litter types, (b) RFP of the two litter types, (c) RCR on unconditioned leaves and the improvement of RCR due to fungal conditioning (Delta RCR), (d) RFP on unconditioned leaves and the improvement of RFP due to fungal conditioning (Delta RFP). Data were log‐transformed (Log *X + 1*) prior the relationship tests. *R* ^2^ of the linear regressions and associated *p*‐values (with the respective symbols *p <* *.05, **p <* *.01, and ***p <* *.001) are indicated

**Figure 5:**
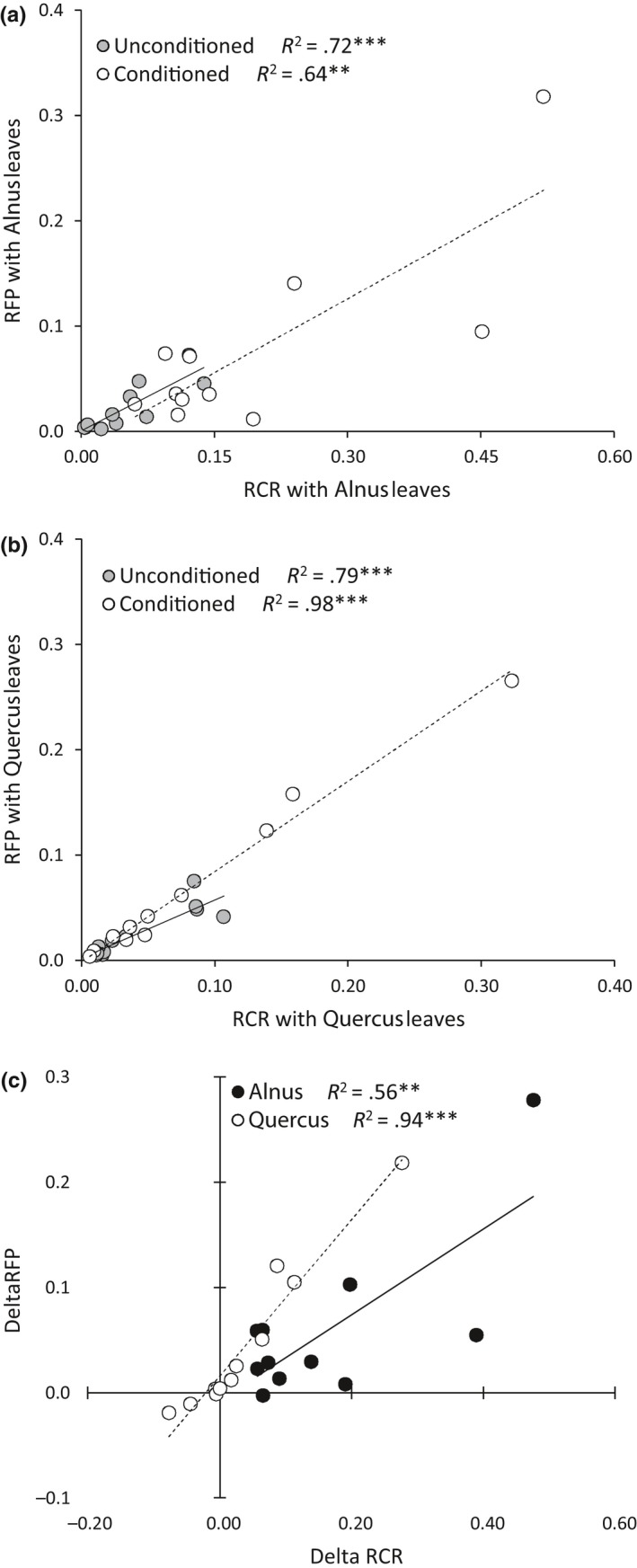
Relationships between (a) RCR and RFP for *Alnus* leaves, (b) RCR and RFP for *Quercus* leaves, (c) the improvement of RCR due to fungal conditioning (Delta RCR) and the improvement of RFP due to fungal conditioning (Delta RFP) for both litter types. Data were log‐transformed (Log *X + 1*) prior the relationship tests. *R* ^2^ of the linear regressions and associated *p*‐values (with the respective symbols **p <* *.01 and ***p <* *.001) are indicated

There was no correlation between RCR for unconditioned leaves of *Alnus* and *Quercus*, whereas there was a strong and positive correlation in RCR between the conditioned leaves of *Alnus* and *Quercus* (Figure 4a). There was a weak positive correlation between the RFP for the unconditioned leaves of *Alnus* and *Quercus*, and a strong positive correlation in RFP between conditioned leaves of *Alnus* and *Quercus* (Figure 4b). The increases in RCR between conditioned and unconditioned leaves were positively correlated to the RCR on unconditioned leaves only for *Alnus* (Figure 4c), suggesting that the more a species consumes *Alnus* litter, the more the microbial conditioning improves its litter consumption. Similarly, the more a species produces feces, the more the microbial conditioning improves its feces production (Figure 4d). RCR and RFP were positively correlated for both litter types (Figure 5a,b). The increases in RCR and RFP between conditioned and unconditioned leaves were positively correlated (Figure 5c), and this relationship was stronger for *Quercus* than for *Alnus*.

RCR and RFP of both litter types were positively correlated to the specialization index only for conditioned leaves. The increases in RCR between conditioned and unconditioned leaves were also positively correlated to the specialization index (Table 5), suggesting that the more a species is specialized in litter consumption, the greater the extent to which microbial conditioning improves its litter consumption.

**Table 5:** Relationships between the specialization index and the litter‐feeding traits according to the litter type (*Alnus* or *Quercus*) and the conditioning level (unconditioned or conditioned leaves). RCR = relative consumption rate, RFP = relative FPOM production, Delta RCR = improvement of RCR due to fungal conditioning, Delta RFP = improvement of RFP due to fungal conditioning. Data were log‐transformed (Log *X + 1*) prior the relationship tests. Adjusted *R* ^2^ in simple linear regressions and associated *p*‐values (with the respective symbols *p <* *.05, and **p <* *.01) are indicated

	Specialization index	
	*Alnus*	*Quercus*
Unconditioned leaves		
RCR	0.22 ns	0.36 ns
RFP	0.36 ns	0.34 ns
Conditioned leaves		
RCR	0.58**	0.54**
RFP	0.42*	0.50**
Delta RCR	0.62**	0.43*
Delta RFP	0.32 ns	0.31 ns

No relationship was observed between AR and RCR, AR and RFP, as well as AR and the specialization index, irrespective of the type of litter and the conditioning level.

## 4 DISCUSSION

This study found considerable interspecific differences in RCR among 11 macroinvertebrate species that differed in terms of taxonomic group, body mass, and specialization level in leaf litter consumption. The RCR found across the 11 macroinvertebrate species was of similar magnitude to those found in previous studies. For example, Prus (1971) and Dehedin, Maazouzi, Puijalon, Marmonier, and Piscart (2013) found respective RCRs of 0.23 and 0.19 mg leaf mg macroinvertebrate^−1^ day^−1^ for *Asellus aquaticus* when feeding on *Alnus glutinosa*, which is close to the 0.21 mg leaf mg macroinvertebrate^−1^ day^−1^ found in this study. Friberg and Jacobsen (1994) found a RCR of 0.13 mg leaf mg macroinvertebrate^−1^ day^−1^, and Dehedin et al. (2013) found an RCR of 0.19 mg leaf mg macroinvertebrate^−1^ day^−1^ for *Gammarus pulex* when feeding on *Alnus glutinosa*. Considering the substantial variation between populations in the consumption rate (Foucreau, Piscart, Puijalon, & Hervant, 2013), the value of 0.17 mg leaf mg macroinvertebrate^−1^ day^−1^ for *Gammarus pulex* in this study is relatively close to these published results. The overall interspecific differences in RCR were negatively related to the macroinvertebrate dry mass, indicating that the consumption rate decreased with organism size. The results of this study are in line with those of Makarievaa et al. (2008), showing that all metazoan groups, including aquatic invertebrates, demonstrate a pronounced decline in mass‐specific metabolic rates with body mass. Strong relationships were also found between RCR and the specialization level in litter consumption for conditioned leaves. This observation hence confirms previous results for native and non‐native amphipods (Piscart et al., 2011) and suggests that specialization increases either the shredding efficiency or the digestion of invertebrates.

As expected, the RCR of macroinvertebrate species was higher when feeding on the high‐quality litter (*Alnus*) rather than poor‐quality litter (*Quercus*), whatever the species. Our results did not highlight a compensatory consumption in the face of low‐quality food. This compensatory consumption was observed with Amphipod and Isopod species in the consumption of aquatic macrophyte *Berula erecta* (Dehedin et al., 2013) and with Diptera and Isopod species (Tyree, Clay, Polaskey, & Entrekin, 2016) in response to an environmental stress. In our experiment, macroinvertebrates generally prefer high‐quality litter and exhibit reduced consumption when forced to feed on low‐quality litter (Cummins & Klug, 1979; Foucreau, Piscart, et al., 2013; Foucreau, Puijalon, et al., 2013; Friberg & Jacobsen, 1994). A low C:N ratio and low phenolic and lignin concentrations in high‐litter quality compared to low‐quality litter could be responsible for the differences in RCR observed in the present study. Indeed, these litter traits are known to control litter breakdown (Assmann, Rinke, Nechwatal, & Von Elert, 2011; Garcia‐Palacios et al., 2016; Gessner, Chauvet, & Dobson, 1999; Ostrofsky, 1997). However, the sensitivity to the leaf litter quality was dependent on the species considered as the increase in RCR ranged from +166% for *Halesus radiatus* to +640% for *Asellus aquaticus*.

The RCR was higher in conditioned leaves compared to unconditioned ones, matching our second hypothesis. The rate of litter consumption by macroinvertebrates is known to be linked to litter colonization by aquatic hyphomycetes (Arsuffi & Suberkropp, 1989; Graça, 2001; Graça, Maltby, & Calow, 1993; Rong, Sridhar, & Barlocher, 1995). Aquatic hyphomycetes could improve the nutritional value of litter by reducing the C:N and C:P ratios (Arsuffi & Suberkropp, 1986, 1989) and decreasing the concentration of secondary compounds, which are known to negatively affect the palatability of leaves (Assmann et al., 2011; Barlocher & Kendrick, 1974). In the present study, a lower C:N ratio and lower phenolic concentrations were observed on conditioned leaves compared to unconditioned ones, which could explain the increase in RCR. Another consequence of the conditioning process by fungi is the reduction in the leaf toughness, which leads to the easier consumption of leaves by invertebrates (Foucreau, Piscart, et al., 2013; Foucreau, Puijalon, et al., 2013; Graça et al., 1993). Another explanation is that invertebrates feed on fungi. Indeed, most of detritivores do not have the enzymatic ability to breakdown the structural compounds of leaves. Fungi and bacteria colonizing leaves produce enzymes able to digest plant cell walls and to liberate simple compounds which can be assimilated by shredders (Foucreau, Piscart, Puijalon, & Hervant, 2016; Graça, 2001). Fungi and Algae growing on leaves themselves may be a feeding target for shredders (Boiché, Gierlinski, & Thiebaut, 2010). All these hypotheses could explain why, as observed for litter quality, the sensitivity to litter conditioning was also species‐specific, as the increase in RCR ranged from +32% for *Crangonyx pseudogracilis* to +479% for *Planorbarius corneus*. Indeed, the relationship between shredders and microorganisms may be the results of many different species‐specific strategies which may explain the variability in their responses.

Contrary to our third hypothesis, the positive conditioning effect found in this study was stronger for high‐quality litter than for low‐quality litter, whereas we thought that the positive consequence of the reduction in leaf toughness should be more important in low‐quality litter (which has tougher leaves). This result suggests that leaf toughness alone is unlikely to be the main factor contributing to the higher litter consumption rate. Interestingly, the positive effect of the conditioning process increased with the level of specialization of the invertebrate, and omnivorous invertebrates (e.g., amphipods and gastropods) remain only weakly affected by the conditioning. The results of this study hence confirmed the strong relationship between aquatic fungi and shredder invertebrates, which likely involves adaptive mechanisms.

The strong correlation in RCR between the two litter types suggests that there is a consistent relationship between RCR and macroinvertebrate species, which is not affected by litter quality. However, microbial conditioning may modulate this relationship, as a strong correlation was only observed with conditioned leaves. Although it is acknowledged that additional litter species need to be tested, this finding suggests that relative differences in litter consumption (namely the rank order in RCR) may not change according to the type of litter.

FPOM production by macroinvertebrates is the predominant food source for many invertebrate species classified as collectors and filter‐feeding invertebrates (Robinson & Minshall, 1990) and also provides strong support for microbial activities (Joyce, Warren, & Wotton, 2007; Joyce & Wotton, 2008; Wallace et al., 1997). As for RCR, FPOM production mainly differed according to macroinvertebrate species. Despite the fact that there is considerable variation in the consumption of litter, its digestion, and the final production of FPOM according to macroinvertebrate species (Arsuffi & Suberkropp, 1989; Bundschuh et al., 2011; Rong et al., 1995), a strong correlation between litter consumption and FPOM production rates was observed. This important finding suggests that RFP could be inferred from RCR, especially for low‐quality litter. The digestion process of leaves in the gut of shredders might also change the quality of the FPOM and hence modify their effect on the wide range of organisms that consume the fecal pellets (Wotton & Malmqvist, 2001). We were not able to determine the mechanism from our methods, and future studies are needed to clarify the species‐specific effects (Joyce et al., 2007). However, the leaf litter material in pellets represents only a small amount of the quality of pellets for which the nutritional value is strongly enhanced by the protein content of colonizing microorganisms (Wotton & Malmqvist, 2001).

Finally, contrary to RCR and RFP, the assimilation rate slightly varied between the 11 macroinvertebrate species and was strongly affected by the litter type. The lower assimilation rate with low‐quality litter (*Quercus*) compared to high‐quality litter (*Alnus*) could be explained by the higher lignin content in *Quercus* litter compared to *Alnus* litter, making leaf assimilation more difficult (Otto, 1974). In contrast to the third hypothesis, the conditioning process did not increase the assimilation of the low‐quality litter. Instead, the conditioning process increased the assimilation of the high‐quality litter, leading to higher decoupling in the assimilation between low‐ and high‐quality litters. This last finding suggests that the conditioning process of high‐quality litter benefits shredders, whereas the conditioning process of low‐quality litter benefits other trophic levels in the detritus‐based food web.

## 5 CONCLUSION

The role played by leaf litter recycling is essential for the functioning of the aquatic ecosystem (Petersen & Cummins, 1974). As demonstrated in this study, the macroinvertebrate species identity is crucial to estimate the effect of the litter quality and microbial conditioning on litter consumption and FPOM production rates. This study showed that leaf consumption and FPOM production rates are mainly dependent on the macroinvertebrate species identity and the conditioning process, whereas the assimilation rate is strongly affected by the litter quality. Macroinvertebrates specializing in leaf litter consumption also appeared to be the most sensitive to shifts in litter quality and the conditioning process. The strong correlation in RCR between the two litter types suggests a consistent relationship between RCR and macroinvertebrate identity that is not affected by litter quality. The RFP was strongly correlated to RCR, suggesting that RFP could be inferred from RCR. Contrary to expectations, the results showed that the conditioning process did not increase the assimilation of low‐quality litter. Our study hence confirms that the relationship between litter, fungi, and invertebrates that are much more complex than currently expected. The varying responses of the macroinvertebrate feeding traits to litter quality and the conditioning process suggest that potential changes in the aquatic macroinvertebrate community could have major consequences for the decomposition process and the detritus‐based food web in streams. In the case of species loss, the identity of the remaining aquatic macroinvertebrates and the knowledge of their associate functional traits would be of great significance to predict the implications for ecosystem processes performed by these species.

## CONFLICT OF INTEREST

None declared.

## AUTHOR CONTRIBUTIONS

MS, LP, and CP conceived and performed the experiments. MS analyzed the data and led the writing of the manuscript. All authors contributed critically to the drafts and gave final approval for publication.
